# The Predictive Utility of Reward-Based Motives Underlying Excessive and Problematic Social Networking Site Use

**DOI:** 10.1177/00332941211025271

**Published:** 2021-06-23

**Authors:** Michael Wadsley, Judith Covey, Niklas Ihssen

**Affiliations:** Department of Psychology, 3057 Durham University, UK

**Keywords:** Social networking sites, social media, reward, motives, addiction, problematic social media use

## Abstract

Compulsive seeking of reward is a hallmark feature of drug addiction, but the role of reward is less well understood in behavioural addictions. The present study investigated the predictive utility of ten reward-based motives, which we identified in the literature, in explaining excessive and problematic use of social networking sites (SNSs). These motives were examined in a cross-sectional survey of 411 young adults, revealing that prolonged use and excessive checking were predicted by distinctly different motives. More frequent checking of SNSs was most closely associated with motives related to obtaining social rewards (impression management/social comparisons/fear of missing out) and the desire to find/consume enjoyable content. In contrast, the amount of time an individual spends on SNSs was predicted by the desire to engage in negative social interactions or to fulfil personal needs (self-expression/documentation of life events). Problematic SNS use was best explained by the motivation to obtain social rewards and to a lesser extent by enjoyment and negative social potency (e.g., trolling) motives. Our results highlight the importance of social reward in explaining excessive and problematic SNS use, suggesting that a focus on reducing the desire to obtain social reward (e.g., through likes, social comparisons, continual connection) may be most beneficial in tackling problematic SNS behaviours.

## Introduction

Social networking sites (SNSs) now play a major role in many people’s day-to-day lives. Especially amongst the younger generation, the use of SNSs has become so ingrained into the daily routine that it forms an integral part of life. Recent estimates indicate that 49% of the world’s population are active social media users, with the average user spending 2 hours 24 minutes on these sites each day (We Are Social, 2020). As technology continues to improve exponentially and the capabilities of SNSs expand it seems only likely that these media will occupy an even more important role in the future. This has led to mounting pressure to understand the consequences that SNS use has on our health and wellbeing, and there is now growing concern regarding its addictive potential (Kuss & Griffiths, 2017). However, in order to better understand compulsive forms of SNS use, we must first understand the individual motivations for SNS use and how they might predict excessive and problematic use.

Within this report we focus specifically on platforms with a primary function of social networking through microblogging or content sharing, in which users can navigate a community-based environment and publicly share personal information whether it be text, image, audio or video, and view content publicly shared by others. Examples of such include Facebook, Instagram, Snapchat, Twitter and YouTube. Although the current literature suffers from a lack of clarity regarding what is and what is not considered a SNS, our focus is consistent with the definition of SNSs as “*virtual communities where users can create individual public profiles, interact with real-life friends, and meet other people based on shared interests*” (Kuss & Griffiths, 2011, p. 3529). While it has been argued by Kuss and Griffiths (2017) that the term SNS is eclectic and encapsulates sites dedicated to gaming (e.g., *World of Warcraft*), dating (e.g., *Tinder*) and instant messaging (e.g., *WhatsApp*) these types of social media were not the focus of this study because their primary function (i.e., instant messaging/dating/gaming) distinguishes them from the microblogging/content sharing platforms previously outlined. Correspondingly, the reward-based motives explaining the use of these platforms might be qualitatively different (e.g., sexual gratification for the use of dating sites) than the motives examined here.

Much discussion currently surrounds the question of whether SNSs (as defined above) can evoke behavioural addictions. Currently the only behavioural addiction formally recognised as such is gambling disorder and while the DSM-V also acknowledges internet gaming disorder as a condition warranting further research, there is no such recommendation for SNS addiction (American Psychiatric Association [APA], 2013a). Different authors have questioned the need for the formal recognition of excessive behaviours as addictions. Some have argued that pathologizing everyday behaviours could damage the relevance and credibility of the addiction field (Kardefelt-Winther et al., 2017). According to this view, researchers are being increasingly led to divert resources towards the study of excessive behaviours that lack the hallmarks of addiction and fail to substantially deviate from normative functioning. As such the validity of the construct ‘behavioural addiction’ is weakened (Billieux et al., 2015; Blaszczynski, 2015). Conversely, others have argued that the similarities between substance addictions and excessive behaviours should not be overlooked (Griffiths, 2017) as there is now accumulating evidence to suggest that some compulsive SNS users display symptoms traditionally associated with substance use disorders (Andreassen, 2015; Kuss & Griffiths, 2011).

There is consensus that substance use disorders are characterised by compulsive seeking and consumption of a chemical substance that directly activates the brain’s reward system and thus evokes pleasure and/or a desire to consume the substance again. This focus on the reward system is reflected in the approach the DSM-V has taken to conceptualise substance-related and addictive disorders in its introductory section (APA, 2013b, p. 481). However, unlike drug addictions in which there is clear evidence that repeated exposure to a rewarding chemical substance results in neural and physiological adaptations that produce physical dependence characterised by withdrawal (Koob & Le Moal, 2008a), reward-related factors that contribute to the development and maintenance of behavioural addictions are less well understood. Thus, in the absence of a psychoactive substance producing neurochemical reward, it is important that we understand the motivational and hedonic incentives behind SNS use. Identifying these antecedents of dysfunctional behaviours is also an essential requirement for the development of targeted interventions.

Previous research has adopted the “uses and gratifications” framework when attempting to identify the motives underlying SNS use (Raacke & Bonds-Raacke, 2008; Whiting & Williams, 2013). However, few studies have considered how the various motives identified in this research might elicit reward or explain problematic SNS use. Focusing specifically on Facebook, Nadkarni and Hofmann (2012) proposed that use is primarily motivated by two basic needs: the need to belong and the need for self-presentation. In a later systematic review of the uses and gratifications of Facebook, Ryan et al. (2014) point to relationship maintenance and passing time as the most important motives underlying its use. However, the spectrum of motives identified by different authors is broad and different studies attribute varying importance to each single motive.

Given the significance of reward for the classification of substance use disorders within the DSM-V, in the present article we consider the capacity of different SNS motives to elicit reward when evaluating their potential to generate excessive and problematic use. Based on a targeted literature review, we identify ten reward-based motives that might explain the development and maintenance of excessive or problematic SNS use. Typically, the term ‘reward’ is used to refer to stimuli or activities that are positive reinforcers based on their incentive properties, that means, their capacity to elicit either pleasure or a motivation (urges or desires) to consume/have the reward (Schultz, 2015). However, given the importance of negative reinforcement for addictive processes (e.g., withdrawal, Koob & Le Moal, 2008b) and the overlap between neural systems underlying positive and negative reinforcement (Schlund et al., 2011), we expand the term ‘reward-based motives’ to refer to motives for SNS use that can be either positive reinforcers (= producing a pleasurable or desired state) or negative reinforcers (= producing a less aversive or undesired state). While not necessarily an exhaustive list, the ten motives identified provide a useful framework for understanding how problematic SNS use might be initiated or maintained. It is likely that individual SNS users possess multiple motives for their use and although we argue that each of the ten reward-based motives represent distinct domains, certain motives may overlap with each other to some extent.

### Impression management

One of the appealing features of conducting social interactions from behind a screen is the control it affords the user in managing how they are perceived. SNSs allow users to easily modify aspects of their identity so that they appear exactly as they wish to be seen by others. Users are able to publicly post content that portrays them as possessing more socially desirable characteristics (e.g., more attractive/healthier/happier) than they might be able to convey in real life interactions. When such actions are affirmed by their peers (e.g., a ‘like’ on Facebook), this elicits a boost in self-esteem and thus a reward (Burrow & Rainone, 2017). Not only does this provide confirmation to the user that their peers approve of their post, it also publicly conveys their popularity to other users who view the post. This social approval may serve to reinforce the use of SNSs in order to maintain favourable appearances and improve one's standing in the social hierarchy. From an addiction perspective, the user may then begin to seek these rewards more frequently and monitor their social acceptability to avoid a drop in self-esteem. In turn, this could result in compulsive checking of the user’s SNS accounts and a behaviour that has been referred to as ‘chasing the like’ (i.e., posting content with the aim of obtaining more and more likes, and deleting content that fails to obtain sufficient likes; La Sala et al., 2015). It should also be noted that such social approval might also be achieved without the need to obtain a ‘like’ for a post. For example, receiving praise through public comments on the post, receiving a friend request or being ‘followed’ or ‘retweeted’ on Twitter or even viewed (e.g., Snapchat provides users with a list of friends who have viewed their story) may produce a similar reward.

A number of studies have reported that seeking attention and acknowledgement from others are primary motives for the use of social media (Stefanone et al., 2011; Sung et al., 2016). Research has also shown that receiving affirmation from peers on content posted on SNSs is associated with increases in self-esteem and subjective wellbeing (Burrow & Rainone, 2017; Oh et al., 2014), yet overreliance on validation from others in pursuit of self-esteem can be costly to well-being in the long-term (Crocker & Park, 2004). In addition, neuroimaging research has shown that viewing photos with many (compared to few) likes resulted in increased activity in brain regions associated with reward processing, social cognition, imitation, and attention (Sherman et al., 2016). For some individuals the reward associated with obtaining a high number of likes may be a significant determinant in their use of SNSs. Research suggests that adolescents and in particular female adolescents may be especially driven to use SNSs because of this motive, as they are more subjected to peer mediation and pressure (Chua & Chang, 2016; Mascheroni et al., 2015).

### Self-expression

Another potential rewarding aspect of SNSs is the ease with which users can clearly express their thoughts, opinions, ideas and beliefs. Regardless of how the content they share is evaluated by others, the user may experience gratification from communicating aspects of their identity. This may be especially important for individuals who might otherwise lack the social skills to communicate aspects of their identity or those who require a wider audience than their immediate social groups in real life (Caplan, 2005). As values related to self-expression have risen in recent decades (Inglehart, 2008; Inglehart & Oyserman, 2004), individuals have increasingly relied on the convenience of online platforms to express themselves (Orehek & Human, 2017).

The rewarding nature of self-expression may be the positive self-affirmation that comes from publicly presenting your true self (Toma & Hancock, 2013). Thus, unlike impression management the use of SNSs for self-expression might be driven by the desire to accurately portray one’s own identity, rather than the desire to obtain positive feedback (e.g., through ‘chasing likes’). Although it seems likely that these two motives might overlap to some extent (i.e., the user may wish for their traits to be viewed both positively and accurately), it is also possible that they manifest independent of each other. Around 30% of everyday conversational speech is devoted to informing others about our own personal experiences (Dunbar et al., 1997) and a content analysis of twitter posts indicates that 41% of all ‘tweets’ consist of announcements about one’s current activities or experiences (Naaman et al., 2010). It therefore seems likely that the need to express information about the self may represent a strong motivational factor in the desire to use SNSs.

It has previously been demonstrated that disclosing information about oneself is strongly associated with increased neural activity in the mesolimbic dopamine system, the same system that is activated by drug and food rewards (Tamir & Mitchell, 2012). Moreover, Tamir and Mitchell (2012) found that individuals are often willing to forgo money in order to disclose information about the self. It is therefore apparent that self-expressing is an inherently rewarding process. Research has also suggested that self-disclosure on SNSs may increase well-being by increasing perceived social support (K. T. Lee et al., 2013).

### Social comparison

Festinger (1954) originally proposed social comparison theory to explain how individuals compare their own opinions and abilities to others in order to generate accurate self-evaluations. Since it was initially proposed research has continued to advance the theory and focus on ways that social comparisons can be used for self-enhancement. Humans show an automatic tendency to evaluate themselves relative to their counterparts (Gilbert et al., 1995; Wood, 1996) and are able to process status cues in others with ease (Zitek & Tiedens, 2012). This serves an obvious evolutionary function. The ability to accurately identify where we stand in a social hierarchy enables us to define social roles and facilitates cooperation (Halevy et al., 2011; Koski et al., 2015). It is well established that social status is strongly associated with self-esteem, wellbeing and health in both humans and animals (Haught et al., 2015; Sapolsky, 2004; Singh-Manoux et al., 2003). The subjective perceptions we form about our own social status will inevitably be influenced by the types of social comparisons we make. It therefore follows that individuals might be motivated to strategically make social comparisons to seek self-enhancement or improve self-esteem.

SNSs offer a unique and unobtrusive means of gathering large amounts of information about the lives of others. Thus, a potential reward-based motive for the use of SNSs might be to make downwards social comparisons with people who are deemed of lower social standing. The concept of downwards social comparisons was first introduced by Wills (1981) who described its basic principle as an attempt to increase one’s subjective self-esteem by making comparisons with a less fortunate other. Thus, individuals might use SNSs to seek information that allows them to make downwards social comparisons generating a rewarding boost in self-esteem. By contrast then it would seem that individuals might avoid upwards social comparisons (i.e., comparing oneself to more fortunate others), as these result in more negative self-evaluations and lower self-worth (Tesser et al., 1988). However, studies have since demonstrated that this is not always the case as individuals can use upwards social comparisons to identify similarities between themselves and the superior other or as a way of gaining inspiration on how to improve (Collins, 1996; Guyer & Vaughan-Johnston, 2018). Thus, it might still be potentially rewarding for individuals to seek upwards social comparisons in an effort to learn how to achieve higher social status. For example, it is possible that individuals might develop compulsive use of SNSs to follow updates from more popular peers or celebrities in order to emulate their behaviours.

In addition, research has shown that using SNSs to make social comparisons is associated with depressive symptoms, and this relationship is particularly strong in females and less popular individuals (Nesi & Prinstein, 2015). For some individuals, making comparisons with others on SNSs may produce a negative cycle of behaviour whereby they attempt to make comparisons for self-enhancement but are unsuccessful in processing the information obtained in a way that enables them to view themselves more positively. A survey of 425 undergraduate students found that those who used social media more frequently were more likely to believe that others were happier and had better lives (Chou & Edge, 2012). Furthermore, Vogel et al. (2014) found that the relationship between chronic SNS use and low self-esteem was mediated by greater exposure to upwards comparisons, and temporary exposure to someone else’s social media profile containing more positive information than one’s own profile (e.g., a high number of ‘likes’ and more healthy life-style) resulted in more negative evaluations of the self. Therefore, social comparisons as a motivation for the use of social media might represent a particularly important indicator of problematic SNS use and negative consequences associated with SNS use.

### Habitual time passing

One commonly reported use of SNSs is passing time (Barker, 2009; Hollenbaugh & Ferris, 2014; Papacharissi & Mendelson, 2011; Smock et al., 2011; Whiting & Williams, 2013). There are many instances throughout the day when it becomes desirable to occupy oneself with an activity in order to pass time. When standing in a queue for example, using smartphone applications can help to ease the tedium of waiting. Research has shown that our sense of time is altered by emotions such that it seems to pass faster when in a state of arousal compared to a drag when bored (Droit-Volet & Meck, 2007). Checking the latest updates on social media is an engaging activity that provides a convenient way of alleviating momentary feelings of boredom.

However, SNSs are designed to capture and hold our attention (Alter, 2017). The more engaging a SNS is the more advertisements are able to be sold, thus generating more revenue for the company. One of the ways that SNSs may encourage repeated use is through the algorithms of the newsfeed page that enable ‘infinite scrolling’ and recommend user specific content. Rather than searching for the content we wish to see; SNS newsfeeds provide a seemingly endless stream of content without a natural stopping point. Such design features have been recognised as encouraging prolonged use, providing a pathway to excessive and problematic SNS use (Montag et al., 2019; Noë et al., 2019) and there have been recent calls for these features to be banned (Hern, 2019). When repeatedly scrolling or refreshing their newsfeeds the user may become lulled into a ‘hypnotic’ state. Such states have become known as ‘ludic loops’ in the context of gambling research and describe a potential mechanism as to how slot machines facilitate compulsive use (Schüll, 2014). Much like slot machines, the very design of a SNS newsfeed creates cycles of uncertainty (i.e., there is always the possibility that the next spin on the slot machine will return a win). When checking their newsfeeds, every so often the user may encounter novel or interesting information that produces a reward. Perhaps they might learn that an old school friend has got married or they will see an interesting news article about a favourite celebrity. However, precisely when the user might encounter an interesting piece of information is often unpredictable, and thus the reward is delivered in what is referred to as random-ratio schedules (Haw, 2008). This uncertainty may reinforce the need to keep checking SNSs as there is the persistent feeling that the next post might be particularly interesting (i.e., highly rewarding). Thus, the user may become locked in a cycle of repeatedly checking SNSs in unconscious anticipation of the next reward, irrespective of whether a reward is actually forthcoming. Once learned, we suggest that the mere process of passing time may become rewarding in and of itself. Consistent with this idea, it has been shown that the anticipation of reward can be a more powerful mediator of addiction than the reward outcome itself, with less predictable outcomes producing greater arousal (Fiorillo et al., 2003; van Holst et al., 2012).

We thus suggest that through these mechanisms, using SNSs as a means to pass time may carry the risk of creating periods of intense, repetitive use behaviour or patterns of ‘mindless’ checking without a specific purpose. A study by Sagioglou and Greitemeyer (2014) found that the negative relationship between Facebook usage and mood was mediated by how meaningful the user believed their activity had been. Accordingly, the habitual use of social media to pass time may likely result in the user feeling that they have achieved less compared to what they might feel when using SNSs with more goal-orientated motives (e.g., to self-express). As a consequence, this motive may be especially salient in problematic users.

### Mood alteration

In contrast to habitual time passing, the motive of mood alteration emphasises deliberate attempts of the SNS user to escape from issues or emotions in the real (offline) world. Sometimes also referred to as ‘escapism’ (Young et al., 2017), mood alteration may facilitate excessive use through negative rather than positive reinforcement: Individuals learn that by using SNSs they can distract themselves from negative affect, such as depressive moods or anxiety.

A number of studies have investigated the relationship between excessive behaviours and the desire to distract oneself from negative emotions. One study comparing the motives of recreational and competitive (esport) gamers found that escapism was a powerful predictor of problematic use in both groups (Bányai et al., 2019). Others have also found an association between problematic social media use and escapism (Brailovskaia et al., 2020; Gao et al., 2017; Kircaburun & Griffiths, 2019; Masur et al., 2014). Escapism is also listed as one of the DSM-V criteria for gambling disorder (APA, 2013b) and different authors have proposed escapism or mood alteration as a clinical marker for gaming (Lemmens et al., 2009), social media (van den Eijnden et al., 2016) and work (Andreassen et al., 2012) addictions. Empirical evidence for the importance of this motive is less clear. For example, Smock et al. (2011) found no association between escapism and Facebook use, while Young et al. (2017) demonstrated that escapism in passive Facebook use (i.e., consuming content) was not associated with Facebook addiction. However, it might be that individuals who use SNSs for mood alteration do so through active use (i.e., communicating with others) and that this is associated with addiction. The contradictory literature on the role of escapism in the use of SNSs casts doubt on the importance of this motive in predicting problematic use and more research is needed to clarify these inconsistencies.

### Fear of missing out

Fear of missing out (FoMO) is defined as “*a pervasive apprehension that others might be having rewarding experiences from which one is absent*”, which evokes a desire to maintain a constant social connection with others (Przybylski et al., 2013, p. 1841). SNSs provide a method to achieve this with a wide network of friends regardless of where they are in the world. Users are able to observe each other’s online activity and keep themselves up to date with the latest events in each other’s lives. In turn, individuals who are particularly orientated towards continual connection with what others are doing may develop feelings of exclusion and anxiety during periods when they are not using SNSs, which continue to build until they check their accounts. Thus, individuals with FoMO may be motivated to use SNSs more frequently in order to alleviate this anxiety which is intensified by their non-use. This desire to be kept ‘in the loop’ may result in compulsive checking behaviours to relieve the anxiety that being ‘out of the loop’ generates, which may have negative and potentially dangerous consequences. For instance, higher levels of FoMO have been shown to be associated with distracted learning and distracted driving as a result of social media use (Przybylski et al., 2013).

While the contribution of FoMO to problematic SNS use is not yet fully understood, this is a topic that has gained considerable attention and there is now a growing body of literature examining the relationship between FoMO and digital technologies. Across multiple cultures FoMO has been shown to correlate with more intense and problematic social media use in adolescents and young adults (Alt, 2015; Beyens et al., 2016; Blackwell et al., 2017; Moore & Craciun, 2020; Oberst et al., 2017; Sheldon et al., 2021; Vaidya et al., 2016). Furthermore, some individuals report using SNSs to create FoMO in others rather than experiencing it themselves (Hetz et al., 2015). Therefore, the use of social media by others may serve to exacerbate FoMO in users who are already predisposed to experiencing fear of social disconnection. Although research has primarily focused on the relationship between FoMO and SNSs in adolescents, some studies have shown that while experiences of FoMO decrease with age, 50% of adults (mean age = 30.8) report experiencing FoMO at least once a month (Milyavskaya et al., 2018). In addition, recent research has found no differences in the levels of FoMO between age cohorts in a sample ranging from 14–47 years old, suggesting that the experience of FoMO may exist independent of age (Barry & Wong, 2020). Therefore, even in older populations, the fear of missing out may represent an important motivation in the desire to use SNSs.

### Relationship maintenance

The need to belong is one of our most basic human needs. Baumeister and Leary’s (1995) influential need to belong theory suggests that humans have an ingrained desire to establish and maintain a minimum quantity of enduring relationships, with frequent non-aversive interactions. This fundamental need is assumed to originate from our tribal past, when belonging to groups was essential for survival (DeWall et al., 2011). While group membership can no longer be considered as essential to surviving in modern society, an unmet need to belong can be detrimental to our health and wellbeing. A sense of belonging is associated with increased self-esteem (Cameron & Granger, 2016) and a lack of belonging has been shown to result in greater instances of depression and suicidality (Fisher et al., 2015; Steger & Kashdan, 2009). Furthermore, neuroimaging research has demonstrated that simulated interactions with friends can activate the brain’s reward circuitry, particularly the striatum and ventro-medial prefrontal cortex (Güroğlu et al., 2008). It is therefore clear to see why maintaining stable relationships is such a powerful and pervasive goal. However, the use of SNSs may fuel the desire to form lasting relationships beyond what might be realistically achieved. Because social interactions with a wide network of individuals are possible through the use of social media, our perception of the extent we can form meaningful bonds with those individuals might become exaggerated.

Early investigations of the uses of SNSs found relationship maintenance to be a primary motive (Raacke & Bonds-Raacke, 2008). This is perhaps unsurprising given that a main function of any social media is to facilitate social interaction. However, the extent to which this motive can be attributed to facilitating problematic use is debateable. While a sizeable body of literature has drawn links between excessive SNS use and lower wellbeing, Clark et al. (2018) suggest that SNSs are beneficial to users when they are used to make meaningful social connections. In their analysis of Facebook communications between 1544 online friendships Sosik and Bazarova (2014) found that frequent and varied Facebook communication predicted the development of stronger relationships while the actual linguistic content of communications did not. Other research has shown that having Facebook friends who are more responsive is more important for satisfying psychological needs than the actual number of Facebook friends one has (Greitemeyer et al., 2014). Thus, individuals may be motivated to engage in excessive and diverse interactions (i.e., likes, comments, tags) to ensure that relationships are strengthened. Through excessive social grooming, users are able to generate a rewarding sense of belonging which may provide another pathway into compulsive use.

### Entertainment

Individuals may also be motivated to use SNSs for entertainment, which can be defined as the intentional consumption of enjoyable content. This motive thus contains a clear pleasure-, and hence reward-seeking component, which might be susceptible to the development of compulsive behaviours similar to other pleasure-evoking activities or substances.

Many previous studies have highlighted the importance of entertainment as a motivation for the use of SNSs. In a survey of YouTube users Khan (2017) found that an entertainment motive was the strongest predictor of the passive consumption of content (i.e., watching videos). Similarly, studies of Facebook users have found entertainment to be the strongest predictor of the intensity of Facebook use (Alhabash et al., 2014; Dhir & Tsai, 2017). However, others have reported entertainment to be a less important motive than using for psychological benefits (e.g., escapism) or social networking (e.g., relationship maintenance; Balakrishnan & Shamim, 2013). While entertainment might be an important motive for SNSs such as Facebook and YouTube, recent research has found no relationship between this motive and compulsive Instagram use (Ponnusamy et al., 2020). Thus, further research is required to establish the role this motive might play in developing problematic use behaviours.

### Archiving

Although SNSs primarily provide a platform to share content amongst friends, they are also commonly utilized as an easy and efficient tool to record our personal life and build personal repositories for meaningful memories (i.e., as a photo album/diary/video diary). Garde-Hansen (2009) describes users’ personal Facebook pages as “*a database of their life, making [it] a collection of collections and collectives*” (p. 141), and Facebook has been recognised as a contemporary way of recording personal identities and histories (Sinn & Syn, 2014). Reflecting on past events through social media may trigger a nostalgic reverie that might reinforce frequent documentation of one’s life. Neuroimaging studies have also linked the experience of nostalgia with activity in the brain’s reward system (Oba et al., 2016). The experience of nostalgia is thought to play an important role in psychological resilience and is positively associated with a sense of meaning in life (Routledge et al., 2011).

Few studies investigating the uses of SNSs have considered archiving as a potential motive. However, the desire to document one’s life has been found to be a primary motive in some studies, especially in the case of Instagram (Sheldon & Bryant, 2016). In a survey of 212 Korean Instagram users, archiving, along with ‘peeking’ (i.e., browsing the photos of others), were shown to be the strongest motivations predicting both positive attitudes towards and intention to use Instagram (E. Lee et al., 2015). The association between Instagram and archiving might be attributed to the fact that Instagram is primarily a site for sharing photos, and self-documentation through images (e.g., selfies) may be a more popular method of archiving than text for example (Sheldon & Bryant, 2016).

However, Sung et al. (2016) has shown that while archiving significantly predicted the intention to post selfies on SNSs, only narcissism – which might be more closely associated with the impression management motive – predicted selfie-posting frequency. This suggests that while archiving may motivate SNS use, it is not necessarily associated with excessive use.

### Negative social potency

Rather than experiencing reward through positive relationships with other SNS users, some individuals may experience reward when engaging in negative online interactions. Consistent with this, individuals with psychopathic traits are less inclined to form meaningful long-term relationships and exhibit atypical experiences of social reward (Foulkes et al., 2014; Mokros et al., 2008; White, 2014). For instance, they may experience prosocial behaviour towards others as less rewarding and derive pleasure from the callous treatment of others (Foulkes et al., 2014). Because SNSs offer a platform to engage in widespread social interactions, individuals who experience reward from antisocial behaviours may be motivated to exploit these functionalities. This is most apparent in ‘trolling’ behaviours, which aim to disrupt or antagonize others online by deliberately posting inflammatory, irrelevant, or offensive content. According to a YouGov survey, as many as 28% of Americans admitted to engaging in troll behaviour by antagonizing a stranger online (Gammon, 2014). As SNSs offer abundant trolling opportunities, it is thus possible that the rewarding nature of these actions may generate compulsive use patterns in some users.

Cheng et al. (2017) argue that under certain circumstances ordinary internet users can become willing to behave like trolls. In their experiment simulating an online discussion, they found that negative mood and seeing troll posts by other users both increased the user’s own trolling behaviour. This suggests that negative social potency might be rewarding even for the average SNS user, when they are in a state of low mood or after witnessing others engage in such behaviour. Offending others or causing harm to others enables individuals to make downward social comparisons with the victim, who is perceived to lose social status through the offense (Wills, 1981). To this extent, the motives of negative social potency and (downward) social comparisons may overlap with each other.

Other research has shown that trolling behaviours are associated with the Dark Tetrad of personality (i.e., narcissism, Machiavellianism, psychopathy, and sadism; Buckels et al., 2014) and in turn, these traits have been associated with more problematic social media use (Kircaburun et al., 2019). However, other research has suggested that reward can be derived from causing social mayhem and that the motivation to do this is a significant predictor of trolling behaviours, above and beyond personality traits (Craker & March, 2016). A recent study assessing the relationship between trait social reward preferences and problematic social media use found that negative social potency was positively correlated with problematic Facebook and Snapchat use (Meshi et al., 2020). Interestingly, of the six social rewards measured, negative social potency was the only reward that produced a significant positive correlation with the problematic use of both platforms. This suggests that the desire to engage in negative online interactions may represent an important indicator of problematic SNS use. However, despite the wide prevalence of trolling, there is currently a lack of research investigating a potential link between negative social potency and compulsive SNS use.

### Present study

The present study sought to investigate the predictive utility of the ten reward-based motives identified in our literature review in explaining excessive and problematic SNS use. Using an online survey, we presented 20 items (two items per motive) to assess the presence of these motives in young adults and their relationship with excessive and problematic SNS use. We predicted that the presence of self-reported reward-based motives (across different types) would be positively associated with frequency of checking SNSs and problematic usage. All items were pre-tested with a sample of 30 participants (28 females, 2 males; mean age: 19.63 years [SD = 2.08]) and pilot data showed adequate associations between motive measures and self-reported SNS usage variables (frequency of checking), with impression management and entertainment potentially showing stronger effects than the other motives. Methods and hypotheses for this study were preregistered on the Open Science Framework website (https://osf.io/jqm57).

## Method

### Participants

An international sample of 411 participants completed the survey (190 male, 214 female, 7 other). The most common nationalities were British (21.4%), Polish (15.3%), Portuguese (11.7%), Italian (5.4%) and Mexican (3.6%). The majority of participants were recruited through external recruitment platforms, such as Polific.co and SurveySwap.io (69.4%), with the rest of the sample being recruited through internal channels or other means (30.6%). Participants received small monetary incentives or course credits for their participation or took part without reimbursement. Participants were aged between 18–30 (M = 22.9, SD = 3.55) and had normal or corrected-to-normal vision. The majority of participants were students (61.6%) and most had completed an undergraduate degree or higher (60%). Data collection took place between April – July 2020. The study was approved by the Ethics Sub-Committee in the Department of Psychology at Durham University and all participants provided fully informed consent.

### Procedure

The survey was set up with PsyToolkit (Stoet, 2010, 2017) and all participants were required to access the survey on a device with a real keyboard and using a browser other than Safari (because of incompatibility with the experiments coded on PsyToolkit). The SNS use behaviour and motive scales were embedded in a larger online study that also included experimental measures not relevant to the current research question, such as reaction-times to SNS logos. The average time taken to complete the entire study was 20.09 minutes. The data which support this publication are available on https://doi.org/10.17605/osf.io/dkr9q.

### Materials

#### Usage intensity

The intensity of SNS use was measured as (a) self-reported daily time spent using SNSs (hours), and (b) the frequency of checking using a 7-point scale (less than daily, daily, every 3–5 h, 2 h, 1 h, 30mins, 15mins). Both usage intensity questions were asked twice, giving separate estimates for usage before and after the COVID-19 virus outbreak. As in this study we were more interested in typical usage behaviour, only the estimates relating to usage frequency before COVID-19 were used.

#### Social media disorder scale

The Social Media Disorder Scale (SMDS; Van Den Eijnden et al., 2016) was used as a measure of problematic SNS use. The scale consists of 9 items based on the DSM-V criteria for Internet Gaming Disorder (Preoccupation, Tolerance, Withdrawal, Persistence, Displacement, Problem, Deception, Escape, Conflict) and uses a dichotomous yes-no scale. Wording of one item (item 9 assessing conflict with parents and siblings in adolescents) was adjusted to make it more appropriate to the age of participants in our sample. Problematic SNS use was measured as the sum of affirmative responses across all items. Scores were not calculated for participants with missing responses on the scale.

#### Reward-based motives

Twenty items were used to assess the ten reward-based motives outlined in the introduction (two items per motive). Each item consisted of a statement (e.g., “I use social media to compare myself to others”) and a 5-item Likert-scale (1 = disagree, 2 = slightly disagree, 3 = neither agree nor disagree, 4 = slightly agree, 5 = agree). Two blocks of 10 items were created with one item for each motive in each block. Block order was randomised across participants. Item order within each block was randomised but constant across participants (see Table 1). The two items per motive were averaged producing ten motive scores that could range from 1–5. Participants were asked to indicate their agreement with each item with regard to their behaviour before the outbreak of COVID-19.

**Table 1.** Items assessing reward-based motives as presented to participants.

Measure	Item
Block 1	
Social comparison	I use social media to compare myself to others.
Archiving	I use social media to document my life.
Impression management	I frequently check social media to see how many likes/retweets my posts have received.
Habitual time passing	I repetitively scroll through social media to pass time.
FoMO	When I don't use social media I experience 'fear of missing out'.
Relationship maintenance	I use social media to maintain my relationships.
Entertainment	I use social media as a source to find entertaining content (e.g., videos/memes).
Negative social potency	I use social media to ‘troll’ others.
Self-expression	I use social media to provide my update/share my opinion.
Mood alteration	I use social media to take my mind off things or calm myself down.
Block 2	
Relationship maintenance	I regularly interact with people on social media to ensure we remain friends.
Impression management	If something I post doesn't get many likes/retweets I will delete it.
Self-expression	I use social media to express my actual self (who I really am).
Negative social potency	I regularly provoke arguments on social media.
Habitual time passing	I often get stuck in a loop of mindlessly checking social media with no real purpose.
Entertainment	I use social media because I can easily search for content that I enjoy.
Archiving	I frequently post content so that I’m able to look back through my life.
Social comparison	I evaluate myself based on other people's social media profiles.
Mood alteration	If I experience negative emotions I will distract myself through social media.
FoMO	I get anxious if I don't check what my friends are doing on social media.

## Results

### Descriptive statistics

The most frequently used SNS in our sample was YouTube (86.4%), followed by Facebook (84.2%), Instagram (83.5%) Twitter (50.1%) and Snapchat (37.5%). Other SNSs used by participants included Reddit (13.1%), TikTok (7.3%) and Tumblr (3.2%). Two participants (0.5%) reported that they did not use a SNS of any kind.

Participants reported spending an average of 3.33 hours (SD = 2.56) on SNSs each day. The median frequency of checking social media was 5 (i.e., once every hour), and the mean SMDS score was 2.01 (SD = 1.64). Thirteen participants with missing data on one or more items in the SMDS were not included in the calculation of the mean SMDS score.

**Figure 1.**
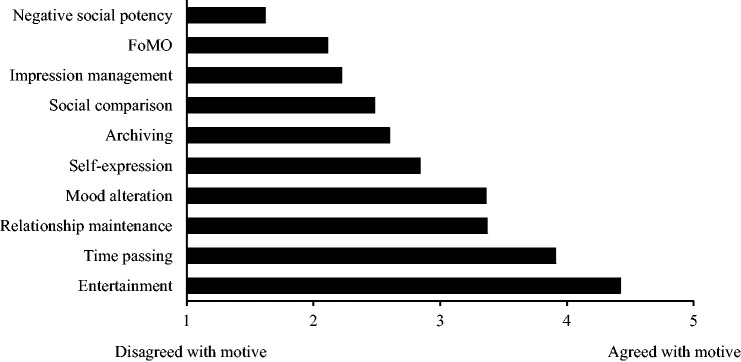
Agreement with ten reward-based motives in the current sample (mean Likert-scale scores). N = 411 for all motives apart from entertainment (N = 410) where an average score could not be calculated for one participant due to missing data.

Mean scores for the ten reward-based motives are displayed in Figure 1. The most strongly agreed with motive for using SNSs was entertainment, followed by time passing, relationship maintenance and mood alteration. The motive with the lowest level of agreement was negative social potency. Cronbach alpha values indicated acceptable reliability for measures of social comparison (*α* = .751), archiving (*α* = .801), relationship maintenance (*α* = .750), negative social potency (*α* = .672), self-expression (*α* = .617) and mood alteration (*α* = .669). However, Cronbach alpha values indicated unacceptable reliability for measures of impression management (*α* = .457), time passing (*α* = .577), FoMO (*α* = .593) and entertainment (*α* = .514). Because of a lack of internal consistency in some of our motive measures we conducted an exploratory factor analysis before entering motives into a regression model.

### Dimension reduction

An exploratory factor analysis using the principal component method with varimax rotation was conducted on the 20 motive items. One item measuring habitual time passing (“I often get stuck in a loop of mindlessly checking social media with no real purpose”) was removed because of comparable factor loadings with factor 1 (.482) and factor 3 (.432). The factor analysis was performed again, and the results of the analysis are displayed in Table 2. Five factors with eigenvalues greater than 1 were extracted and they collectively accounted for 58.48% of the variance of the original item variables. Cronbach α values for each factor ranged from 0.66 to 0.78 indicating acceptable reliability. Factor 1 contained 6 items consisting of both measures/items of social comparison, FoMO and impression management. These items are related to rewards obtained by (actively or passively) interacting with others, either by comparing oneself to another, obtaining ‘likes’ from another or by staying connected with what others are doing. Therefore, we named this factor ‘social reward’. ‘Social reward’ was associated with the highest eigenvalue among all 5 factors. Factor 2 contained 4 items consisting of both measures of archiving and self-expression. These items are related to personal motivations for SNS use where the reward is obtained by fulfilling one’s own goals (i.e., expressing personal views and looking back on past events). Therefore, we named this factor ‘personal utility’. Factor 3 contained 5 items consisting of both measures of entertainment and mood alteration and one measure of time passing. These items all relate to the use of SNSs for enjoyment purposes (associated with accessing specific SNS content), either to escape negative emotions, pass time or for entertainment. We therefore named this factor ‘enjoyment reward’. Factors 4 and 5 contained only two items each, consisting of both items related to a single motive (negative social potency or relationship maintenance). As such, factor 4 was named negative social potency and factor 5 was named relationship maintenance.

**Table 2.** Factor analysis using varimax rotation of the reward-based motives to use SNS.

Factors	Loadings
*Factor 1: Social reward* (*α* = .76, M = 2.27, SD = 0.88)	
Social comparison (B1)	.794
Social comparison (B2)	.794
FoMO (B1)	.578
FoMO (B2)	.567
Impression management (B1)	.545
Impression management (B2)	.516
Variance (eigenvalue)	15.31 (2.91)
*Factor 2: Personal utility* (*α* = .78, M = 2.72, SD = 1.14)	
Archiving (B2)	.790
Archiving (B1)	.788
Self-expression (B2)	.699
Self-expression (B1)	.668
Variance (eigenvalue)	13.66 (2.60)
*Factor 3: Enjoyment reward* (*α* = .66, M = 3.94, SD = 0.72)	
Entertainment (B1)	.694
Mood alteration (B1)	.654
Time passing (B1)	.640
Entertainment (B2)	.611
Mood alteration (B2)	.589
Variance (eigenvalue)	11.50 (2.19)
*Factor 4: Negative social potency* (*α* = .67, M = 1.63, SD = 0.98)	
Negative social potency (B2)	.829
Negative social potency (B1)	.807
Variance (eigenvalue)	9.50 (1.81)
*Factor 5: Relationship maintenance* (*α* = .75, M = 3.37, SD = 1.27)	
Relationship maintenance (B1)	.875
Relationship maintenance (B2)	.822
Variance (eigenvalue)	8.50 (1.62)

*Note*. B1 refers to the item in block 1 whereas B2 refers to the item in block 2.

### Regression analysis

Because the exploratory factor analysis reduced our 10 proposed motives to 5 factors with acceptable internal consistency, we ran our pre-registered stepwise regression analyses for each dependent measure (time spent using SNSs, frequency of checking SNSs, and SMDS score) using the 5 variables generated from the factor analysis. Results are shown in Table 3. Before running our regression analyses potential gender differences in our dependent measures were explored using independent *t*-tests. Males (M = 3.46, SD = 2.79) and females (M = 3.25, SD = 2.37) did not significantly differ in their self-reported time spent using social media [*t*(402) = 0.82, *p* = .414]. Nor did males (M = 4.41, SD = 1.48) and females (M = 4.52, SD = 1.51) differ in their self-reported frequency of checking [*t*(402) = −0.79, *p* = .429]. However, females (M = 2.28, SD = 1.71) did score significantly higher than males (M = 1.73, SD = 1.52) on the SMDS, indicating more problematic use [*t*(389) = −3.32, *p* = .001]. Because of this significant gender difference, the gender variable was dummy coded and included in our regression model to predict SMDS score. Assumption checks revealed no evidence of outliers, multicollinearity or heteroscedasticity.

**Table 3.** Results of the stepwise regression analyses using factors generated from the factor analysis to predict daily time spent using SNSs, frequency of checking SNSs and SMDS score.

Variables	*B*	*SE B*	*β*	*p*
Time spent using				
Negative social potency	0.451	0.127	.173	<.001
Personal utility	0.367	0.110	.163	.001
Frequency of checking				
Social reward	0.427	0.083	.251	<.001
Enjoyment reward	0.297	0.101	.143	.004
Problematic use (SMDS score)^a^				
Social reward	0.653	0.085	.353	<.001
Enjoyment reward	0.422	0.103	.182	<.001
Negative social potency	0.364	0.078	.217	<.001
Female	0.403	0.154	.123	.009

^a^Thirteen participants were excluded from the analysis because of missing data on one or more items in the SMDS.

The model for daily time spent using SNSs was significant [*F*(2, 408) = 14.75, *p* < .001] with an *R*^2^ = .067. The only two significant predictors were negative social potency and personal utility, which both predicted greater SNS use. The stepwise regression of SNS checking frequency revealed two different significant predictors: social reward and enjoyment reward, *F*(2, 408) = 23.20, *p* < .001, *R*^2^ = .102, with social reward showing a substantially higher regression coefficient (see Table 3). Finally, the 5 factors and dummy coded gender variables (male, female and other) were entered into a stepwise regression analysis to predict problematic use (SMDS scores). The four significant predictors included in the model were social reward, enjoyment reward, negative social potency and female, *F*(4, 392) = 42.84, *p* < .001, *R*^2^ = .304, with social reward showing the highest beta.

Together, our results suggest that gender and reward-based motives might better explain problematic social media use (accounting for 30.4% of the variance) than excessive use (accounting for 6.7% of the variance in time spent using and 10.2% of the variance in frequency of checking). Moreover, both the frequency of checking and problematic use appear to be most strongly determined by social reward, and to a lesser extent by the desire to find enjoyable content (enjoyment reward). Notably, our results also indicate an important role of antisocial motives (negative social potency) in predicting problematic use behaviour and prolonged time spent on SNSs.

## Discussion

The present study sought to investigate the predictive utility of ten reward-based motives in explaining excessive and problematic SNS use. Consistent with previous research, descriptive statistics showed that on average entertainment, time passing, and relationship maintenance were most salient when participants self-rated the presence of different motives (Ryan et al., 2014). Interestingly however, when predicting actual use behaviour (quantity and problematicity), other types of motives also played a role.

Based on an analysis of internal consistency of our 10 original motive measures, we conducted a factor analysis that reduced our ten motives to five factors, each with acceptable reliability. The reduction from 10 to 5 constructs confirmed our initial suspicion (see introduction) that some concepts identified in our literature review overlapped with each other to some extent. The 5 extracted factors were labelled ‘social reward’ (consisting of social comparison, FoMO and impression management motives), ‘personal utility’ (archiving and self-expression motives), ‘enjoyment reward’ (entertainment, mood alteration and time passing motives), ‘negative social potency’ and ‘relationship maintenance’.

Using the predictor variables generated from the factor analysis, we then ran our preregistered stepwise regression analyses. These analyses revealed that daily time spent using SNSs and frequency of checking were associated with distinctly different motives. More prolonged SNS use was associated with the factor ‘personal utility’ (for self-expression/archiving) and the motivation for engaging in negative social interactions (e.g., trolling). Conversely, more frequent checking was associated with the factor ‘social reward’ (e.g., gaining social approval/making comparisons with others/maintaining continual social connection) and the desire to find and consume enjoyable content (‘enjoyment reward’). These differences in the motives that predict frequent checking versus prolonged use suggest a behavioural dissociation between manifestations of excessive SNS use that might warrant further investigation. If excessive SNS use is to be considered a marker of behavioural addiction, then the distinction between excessive time spent using and excessive checking may be an important aspect of determining what constitutes problematic use. As our findings indicate that the motives predicting checking frequency more closely resemble the motives that predict problematic use, it might be the case that compulsive checking represents a more important indicator of addiction than the actual duration an individual user spends on SNSs.

Consistent with this idea, our results show that the motivation to obtain social rewards has not only an important relationship with checking frequency but also in explaining problematic SNS use (SMDS score). Indeed, the factor ‘social reward’ (consisting of the average score of the items measuring impression management, social comparison and FoMO) was the strongest predictor of both measures, underscoring the significance of social reward processes for SNS behaviours more generally. Our finding adds to the growing recognition of social reward as being a fundamental driver of human behaviour, similar to non-social rewards (Bhanji & Delgado, 2014). The important influence of approval, acceptance and other social rewards on behaviour is also demonstrated by neuroimaging work, showing that being liked and accepted by others activates similar brain regions as those that are activated by powerful non-social rewards, such as money or food (Davey et al., 2010; Fareri & Delgado, 2014). Our finding is also consistent with previous research suggesting that addictive SNS use reflects a need to feed the ego and inhibit negative self-evaluations (Andreassen et al., 2017). Thus, the boost in self-esteem associated with gaining social approval (Burrow & Rainone, 2017), the temptation to engage in social comparisons (Chua & Chang, 2016) and the desire to maintain continual connection with what others are doing (Przybylski et al., 2013) may all play an important role in facilitating compulsive SNS use. In turn, in ‘healthy’ SNS users the presence of desires to excessively engage in these behaviours might represent useful indicators of risk factors for developing problematic use. Interventions that aim to reduce the motivation to gain approval and make comparisons on SNSs, might therefore be most beneficial in reducing problematic use behaviours. For example, Instagram has recently trialled removing the ability to view the ‘like’ count on other people’s posts in some countries (BBC News, 2019). Although this trial has only been conducted on one social media platform and is restricted to a few countries, recent research has suggested that the decision has been well-received by Instagram users with the majority reporting that removing the ability to view likes on social media would improve mental health (e.g., by reducing validation anxiety; Prichard et al., 2021).

The regression analyses also showed that, to a lesser extent, checking frequency and SMDS score were predicted by the factor ‘enjoyment reward’. Therefore, individuals who excessively use SNSs as a means to find and consume pleasure-inducing content - either to escape, pass time or for entertainment - may also be at risk of developing problematic use behaviours. Compulsive use of SNSs for enjoyment purposes may be facilitated by the inherent properties of SNSs – offering uncertain reward and creating a more or less permanent state of reward anticipation, similar to slot machines (Schüll, 2014) – ultimately leading to excessive checking behaviours. These unpredictable patterns of reward delivery (i.e., random-ratio schedules) have long been understood to be highly engaging compared to more predictable schedules of reward (Ferster & Skinner, 1957). Furthermore, random-ratio schedules have been shown to maximize the release of dopamine in the midbrain and parts of the basal ganglia known to be involved in reinforcing reward seeking behaviour (Fiorillo et al., 2003; Zald et al., 2004). As outlined in the introduction, there are specific SNS features that promote unpredictable reward experiences, such as newsfeeds that enable infinite scrolling. Many SNS newsfeeds are constantly updated with new content and offer no natural stopping point, making continual scrolling or persistent checking in anticipation of the next reward a highly engaging activity. Thus, interventions that place caps on the amount of content that can be viewed through a user’s newsfeed over a specified period of time may have certain utility in the same way that setting voluntary bet limits can help intense online gamblers control their betting behaviour (Auer & Griffiths, 2013). Taken together, our results support the findings of recent research which found that time passing, socializing, presenting a more popular self and entertainment motives all predict more problematic social media use (Kircaburun et al., 2020).

An unexpected finding of our study was that negative social potency significantly predicted SNS use duration and SMDS score. Few studies have investigated negative social potency as a motivation for using social media, and fewer still have investigated its association with excessive or problematic use. Nonetheless, a recent study corroborates our finding, showing that a motive to cause social mayhem online predicts problematic SNS use (Meshi et al., 2020). In their study the authors correlated scores on the Social Reward Questionnaire (SRQ; Foulkes et al., 2014) with scores on measures of Facebook and Snapchat addiction. Interestingly, of the six social rewards measured only negative social potency was correlated with more problematic use across both platforms. The researchers suggest that individuals with a motivation to be cruel and callous to others might be more likely to repeatedly engage in negative online behaviours, such as trolling and cyberbullying. The abundant opportunities that SNSs offer to engage in these behaviours might reinforce problematic use and provide a pathway to addiction. We suggest that future research should explore the potential relationship between negative social potency and problematic social media use further as there is currently a lack of research explaining how this motive might facilitate SNS addiction.

Interestingly, despite predicting prolonged use, the factor ‘personal utility’ did not predict problematic SNS behaviours. ‘Personal utility’ was constructed by combining the motive measures of self-expression and archiving. The reward underlying this motive can be described as a positive self-affirmation derived from expressing one’s true self and/or documenting one’s life. Possessing motives for self-expressions and archiving seems to encourage extended periods of use. However, research on the importance of this motive remains scarce and it seems that an archiving/self-documentation motive may be more important for certain platforms, such as Instagram (Sheldon & Bryant, 2016). While some studies have shown that self-expression and self-documentation motives predicted more intense Facebook use (Alhabash et al., 2014), others have found that a motive to use SNSs as a task management tool (e.g., to store and organise photos) did not predict social media use (Kircaburun et al., 2020). However, our data suggest that while a personal utility motive may promote prolonged SNS use, individuals with this motive are less likely to report using SNSs problematically.

While on average ‘relationship maintenance' was the third most popular motive for SNS use in our sample, it did neither explain excessive nor problematic use. As outlined above, this motive can be considered as a manifestation of the need to belong (Baumeister & Leary, 1995), whereby users seek frequent, diverse and reciprocal interactions with friends online. The lack of association between the relationship maintenance motive and problematic SNS use is in line with research showing that the use of SNSs may have positive effects on wellbeing when they are used to make meaningful connections (Clark et al., 2018). This suggests that using SNSs for relationship maintenance motives may represent a ‘healthier’ use behaviour than using SNSs for other, more self-related purposes, such as gaining approval or social comparisons.

Finally, while males and females did not significantly differ from each other in regard to their self-reported usage intensity, we did observe that females scored significantly higher than males on the SMDS indicating more problematic use. We therefore controlled for this gender difference in our regression model predicting SMDS score. Our results are consistent with findings showing that females are more likely to display higher levels of SNS addiction whereas males are more prone to developing an internet gaming disorder (Su et al., 2020).

### Limitations

Similar to other survey-based research, the above findings are limited by biases inherent in self-report measures, such as socially desirable responding and self-consistency (Podsakoff et al., 2003). Further, despite a comprehensive review of the existing literature the list of motives investigated in our study is non-exhaustive and it is possible that other motives may also play a role in explaining SNS behaviours. Identifying new motives underlying SNS use in future studies is especially important insofar as social media technologies will continue to evolve and diversify over time, limiting the temporal validity of our findings. The cross-sectional design also limits the ability to make causal inferences and consequently the direction of the reported effects cannot be determined (i.e., certain motives may be the consequence of more problematic use). Thus, more longitudinal research is needed to ascertain the directionality of the relationship between reward-based motives and excessive/problematic SNS use. Finally, while our study used a multinational sample, the age range was restricted to young adults, with a majority of participants being university educated students. Previous studies have shown a moderating role of age and other sociodemographic variables on SNS use behaviours (Andreassen et al., 2017; Rumpf et al., 2014; Su et al., 2020). It thus seems important to examine differences in reward-based motives contingent on such variables in future research.

## Conclusion

Taken together, our findings provide evidence for the importance of reward-based motives in determining the intensity of SNS use but also in explaining compulsive or problematic use behaviours. In general, reward-based motives appear to predict problematic use (SMDS score) more accurately (with regard to explained variance) than use intensity (checking frequency and time spent on SNSs). Our data also suggest that distinct motives are associated with the frequency of checking SNSs and the actual use duration. Importantly, a high motivation to obtain ‘social rewards’ (e.g., through social approval, continual connection and social comparison) is the most important indicator of excessive checking and problematic SNS use. The pivotal role of social rewards for SNS behaviour corroborates the notion that social and non-social reward signals converge on a common brain system that guides human behaviour in a diverse range of contexts (Fareri & Delgado, 2014). Given the importance of social reward for SNS use, our results suggest that interventions that target social reward processes (such as removing the visibility of ‘likes’) may offer the most promising avenue to reduce compulsive SNS use.
